# Admission systemic inflammatory indices and 6-month functional outcome after endovascular thrombectomy for acute ischemic stroke: an exploratory single-center cohort study

**DOI:** 10.3389/fneur.2026.1819306

**Published:** 2026-08-04

**Authors:** Xiang Fang, Wei-fen Chen, Juan Wu, Taijian Liao, Chengmin Tong, Biyu Xu

**Affiliations:** 1 Division 1, Neurology Department, Nanping First Hospital Affiliated to Fujian Medical University, Nanping, Fujian, China; 2 Nanping First Hospital Affiliated to Fujian Medical University, Nanping, Fujian, China

**Keywords:** acute ischemic stroke, endovascular thrombectomy, functional outcome, neutrophil-to-lymphocyte ratio, stroke severity, systemic immune-inflammation index

## Abstract

**Background:**

Many patients still have poor functional outcomes after endovascular thrombectomy (EVT) for acute ischemic stroke. Systemic inflammatory indices from routine blood tests have been proposed as accessible prognostic biomarkers. We evaluated the association of admission neutrophil-to-lymphocyte ratio (NLR), platelet-to-lymphocyte ratio (PLR), systemic immune-inflammation index (SII), and systemic inflammation response index (SIRI) with 6-month functional outcome after EVT, and tested whether they provide incremental prognostic value beyond the National Institutes of Health Stroke Scale (NIHSS).

**Methods:**

We retrospectively analyzed consecutive patients undergoing EVT for anterior circulation acute ischemic stroke at one center. The primary outcome was poor functional outcome (modified Rankin Scale [mRS] 4–6) at 6 months. Associations were assessed with logistic regression and ROC analysis. Incremental value over NIHSS was assessed with likelihood-ratio tests, AUC comparison (DeLong), reclassification metrics (NRI, IDI), and decision-curve analysis.

**Results:**

Of 90 patients, 82 (91.1%) completed follow-up, of whom 40 (48.8%) had poor outcomes; regression used 75 patients with complete inflammatory-index data (35 events). At the univariable level, poor outcome was associated with higher NIHSS (median 15.5 vs. 8.0, *p* < 0.001), NLR, SII, and SIRI (all *p* < 0.05). In multivariable analysis, only age was independently associated with poor outcome (OR 1.09, 95% CI 1.02–1.16, *p* = 0.008); the inflammatory indices were not, reflecting strong collinearity with NIHSS (*r*_s_ = 0.88). NIHSS showed the highest discrimination (AUC 0.754, 95% CI 0.636–0.858), followed by SII (0.716, 0.592–0.836) and NLR (0.695, 0.570–0.808). Adding any inflammatory index to NIHSS did not improve discrimination or reclassification (likelihood-ratio *p* ≥ 0.40; ΔAUC ≤ 0.006; IDI ≈ 0; no meaningful net benefit), and results were unchanged under multiple imputation, though the sample could not exclude a small effect.

**Conclusion:**

Admission NLR, SII, and SIRI are associated with 6-month poor outcome after EVT, but this association is largely explained by stroke severity, with no detectable incremental value beyond NIHSS. These exploratory findings are hypothesis-generating and require prospective multicenter validation before clinical use.

## 1 Introduction

Acute ischemic stroke (AIS) due to large vessel occlusion (LVO) remains a devastating cerebrovascular event associated with high rates of disability and mortality (1). Multiple landmark randomized controlled trials have established endovascular thrombectomy (EVT) as the standard of care for eligible patients with anterior circulation LVO (2, 3). A pooled individual-patient-data meta-analysis of five pivotal trials (HERMES) reported a number needed to treat of 2.6 for reduced disability (4). Yet approximately 40%–55% of EVT-treated patients still experience poor functional outcomes (mRS 4–6) at 3–6 months, underscoring the need for early prognostic stratification (5, 6).

Systemic inflammation plays a pivotal role in the pathogenesis and progression of ischemic brain injury (7). Following arterial occlusion, neutrophil infiltration, blood–brain barrier disruption, platelet activation, and lymphocyte-mediated immunosuppression collectively exacerbate injury and influence recovery (8). Hematological indices derived from the routine complete blood count (CBC)—NLR, PLR, SII (platelet × neutrophil/lymphocyte), and SIRI (neutrophil × monocyte/lymphocyte)—are practical, low-cost surrogates of this systemic inflammatory state (9, 10), and have prognostic value across malignancies and cardiovascular disease (11).

In AIS, individual inflammatory indices have been associated with short-term outcomes, but evidence in EVT-treated patients remains limited and inconsistent (12). Two specific gaps motivate the present study. First, few studies have compared NLR, PLR, SII, and SIRI head-to-head within the same EVT cohort against a 6-month outcome. Second, and more importantly, prior reports have generally emphasized the statistical *association* of these markers with outcome without formally testing whether they add prognostic information incremental to the established clinical predictor, NIHSS, with which they are biologically and statistically intertwined (13).

We therefore conducted an exploratory analysis with a deliberately critical aim. We hypothesized that admission inflammatory indices are associated with 6-month poor outcome at the univariable level, but that this association is largely mediated by stroke severity (NIHSS) and provides little or no incremental discriminative value once NIHSS is accounted for. Rather than assume independent predictive utility, we set out to quantify it directly.

## 2 Methods

### 2.1 Study design and population

This single-center retrospective observational cohort study included consecutive patients who underwent EVT for anterior circulation AIS between January 2022 and December 2024 at Nanping First Hospital Affiliated to Fujian Medical University. The protocol was approved by the Medical Ethics Committee of Nanping First Hospital (Approval No.: NPSY2021120010); individual informed consent was waived owing to the retrospective design. The study followed the Declaration of Helsinki (2013 revision) and is reported per the STROBE guideline. No a priori sample-size or power calculation was performed; this was a convenience sample comprising all consecutive eligible patients during the study period, and the resulting limitations in statistical power are addressed in §4.5.

Inclusion criteria were: (1) age ≥ 18 years; (2) anterior circulation AIS with LVO (ICA terminus, MCA M1 or M2) confirmed by CTA/MRA and DSA; (3) treatment with EVT (stent retriever and/or aspiration, with or without bridging thrombolysis); (4) EVT initiated within 24 h of onset/last-known-well; and (5) admission blood sampling as defined in §2.4.

Exclusion criteria were: (1) posterior circulation stroke; (2) pre-existing hematological disorders; (3) active malignancy or chemotherapy within 6 months; (4) concurrent systemic infection at presentation; (5) chronic immunosuppressive therapy; (6) pre-stroke mRS ≥ 3; and (7) incomplete baseline hematological data precluding index calculation.

### 2.2 EVT procedure

All procedures were performed by experienced interventional neuroradiologists under general anesthesia or conscious sedation. Technique (stent retriever, ADAPT, or combined) was at operator discretion; a maximum of five passes was permitted. Angiographic outcomes were assessed on the final DSA run by two independent neuroradiologists blinded to clinical outcomes, with discrepancies resolved by consensus.

### 2.3 Data collection and variable definitions

Baseline demographics and clinical characteristics were extracted from electronic medical records by trained coordinators. Stroke severity was assessed by certified stroke neurologists using NIHSS. Recanalization was graded with the modified Thrombolysis in Cerebral Infarction (mTICI) scale; for analysis, grades were collapsed into three ordinal categories: mTICI 1 (failure, 0–2a), mTICI 2b, and mTICI 3 (2c–3). Successful recanalization was defined as mTICI 2b–3.

### 2.4 Hematological parameters and inflammatory indices

Admission hematological parameters (neutrophil, lymphocyte, platelet, and monocyte counts) were obtained from the first CBC drawn on arrival to the emergency department, prior to administration of any thrombolytic or anticoagulant agent and prior to EVT. Samples were analyzed on a Sysmex XN-Series analyzer. NLR, PLR, SII, and SIRI were calculated as defined above and treated as continuous variables. We acknowledge (§4.5) that single admission sampling cannot capture the dynamic inflammatory trajectory.

### 2.5 Outcome assessment

The primary outcome was poor functional outcome (mRS 4–6) at 6 ± 1 months. We selected the mRS 4–6 threshold because it identifies patients dependent for basic bodily needs or deceased, a clinically critical state, and because in our high-severity EVT cohort (median NIHSS 12) it yielded a clinically meaningful and well-balanced split (48.8% events); the more common mRS 0–2/0–3 dichotomies would have produced more imbalanced groups in this population. As a sensitivity check we confirmed that the direction of associations was consistent under the mRS 0–2 vs. 3–6 split. Outcomes were assessed by two trained neurologists blinded to laboratory values (weighted kappa > 0.80). Patients unreachable after ≥ 3 attempts were classified as lost to follow-up.

### 2.6 Statistical analysis

Analyses were performed in R 4.3.3. Continuous variables were assessed for normality (Shapiro–Wilk, Q–Q plots); non-normally distributed variables are reported as median (IQR) and compared with the Mann–Whitney *U* test, and normally distributed variables as mean ± SD and compared with Student’s *t*-test. Categorical variables are reported as *n* (%) and compared with the *χ*^2^ or Fisher’s exact test.

Univariable logistic regression was performed for each candidate predictor. Variables with *p* < 0.10 univariably, plus *a priori* clinical variables (age, sex), entered a multivariable model (enter method). Because NLR, SII, SIRI, and NIHSS are strongly collinear (sharing stroke severity as their upstream driver), we structured the modeling to make this collinearity explicit rather than to assert independent effects. PLR was excluded from multivariable modeling as a mathematical subcomponent of SII with severe collinearity. Multicollinearity was quantified by the variance inflation factor (VIF; VIF > 5 considered problematic), reported with the model from which each VIF derives. Model calibration was assessed by the Hosmer–Lemeshow test.

The primary multivariable model (Model 1) included age, sex, NIHSS, mTICI, NLR, and SII. Model 1 is presented to quantify the extent to which the inflammatory signal is subsumed by NIHSS, not to establish independent effects, whose coefficients are unreliable under this degree of collinearity. A sensitivity model excluding NIHSS is reported in §3.5 with an explicit caveat that it cannot establish independence (see §4.3). Given the low events-per-variable ratio (35 events/six predictors ≈ 5.8), we additionally fitted a Firth penalized logistic regression as a robustness check, and we performed a multiple-imputation sensitivity analysis (*m* = 25, all 82 patients with outcome data, chained-equation imputation, Rubin’s rules), with results cross-checked using an independent implementation (R mice).

Discrimination was assessed by ROC analysis, with AUCs reported with bootstrap percentile 95% CIs (2,000 resamples) and compared pairwise using the DeLong test; optimal cutoffs were derived by the Youden index. To assess incremental value over NIHSS, we (i) compared nested models (NIHSS vs. NIHSS + each index) by likelihood-ratio test and AIC; (ii) compared their AUCs by the DeLong test; (iii) computed category-free NRI and IDI; and (iv) performed decision-curve analysis across threshold probabilities of 0.10–0.90. Spearman correlations quantified the association of NIHSS with each index. Trend across mTICI grades was assessed with the Jonckheere–Terpstra and Cochran–Armitage tests. A two-sided *p* < 0.05 was considered significant; secondary analyses are exploratory and unadjusted for multiplicity.

## 3 Results

### 3.1 Study population

Of 112 patients screened, 22 were excluded (posterior circulation, *n* = 9; active malignancy, *n* = 3; concurrent infection, *n* = 4; pre-stroke mRS ≥ 3, *n* = 3; incomplete laboratory data, *n* = 3), leaving 90 enrolled. Of these, 82 (91.1%) completed 6-month follow-up and 8 (8.9%) were lost to follow-up (non-differential with respect to baseline NIHSS, *p* = 0.75, and age, *p* = 0.24). Regression, ROC, and correlation analyses used the 75 patients with complete data for all four inflammatory indices (35 events; this uniform analytic sample was adopted so that all models in Table 1 are estimated on identical patients). Median age was 70.0 years (IQR 65.0–76.0); 50 (61.0%) were male; median NIHSS was 12.0 (7.0–18.0). Successful recanalization (mTICI 2b–3) was achieved in 71/82 (86.6%).

**Table 1:** Logistic regression for poor functional outcome (mRS 4–6) at 6 months (*n* = 75).

Variable	Univariable OR (95% CI)	*p*	Multivariable OR (95% CI)^1^	*p*	VIF^2^
Age, per year	1.07 (1.01–1.13)	0.013	1.09 (1.02–1.16)	0.008	1.04
Male sex	0.72 (0.28–1.86)	0.500	0.42 (0.13–1.32)	0.138	1.07
NIHSS, per point	1.14 (1.05–1.22)	<0.001	1.18 (0.98–1.42)	0.087	5.64
mTICI, per grade	0.61 (0.30–1.25)	0.176	0.57 (0.24–1.35)	0.201	1.03
NLR, per unit	1.85 (1.21–2.83)	0.005	1.11 (0.28–4.35)	0.885	8.66
SII, per 100 units	1.20 (1.04–1.38)	0.010	0.93 (0.68–1.27)	0.653	4.20

1 Model 1 (enter method); Hosmer–Lemeshow *p* = 0.247.

2 VIF from Model 1; values > 5 (NIHSS, NLR) indicate problematic collinearity. A sensitivity model excluding NIHSS, and Firth penalized and multiple-imputation analyses, are described in §3.5 and §3.8.

### 3.2 Functional outcomes

At 6 months, 42 patients (51.2%) had good (mRS 0–3) and 40 (48.8%) had poor (mRS 4–6) outcomes. The mRS distribution was: 0, 6 (7.3%); 1, 7 (8.5%); 2, 10 (12.2%); 3, 19 (23.2%); 4, 17 (20.7%); 5, 10 (12.2%); 6, 13 (15.9%). Six-month mortality (mRS 6) was 15.9%.

### 3.3 Baseline characteristics by outcome

As shown in Table 2, patients with poor outcomes were older patients with poor outcomes were older (median 71.0 vs. 67.5 years, *p* = 0.033) and had markedly higher NIHSS (15.5 vs. 8.0, *p* < 0.001). They showed a pro-inflammatory profile: higher neutrophils (*p* = 0.003), lower lymphocytes (*p* = 0.003), and higher NLR (*p* = 0.003), PLR (*p* = 0.008), SII (*p* = 0.001), and SIRI (*p* = 0.023). Sex, BMI, mTICI distribution, platelet count (*p* = 0.27), and monocyte count (*p* = 0.90) did not differ significantly.

**Table 2:** Baseline characteristics stratified by 6-month functional outcome (*n* = 82).

Variable	Total (*n* = 82)	Good (*n* = 42)	Poor (*n* = 40)	*p*
Demographics				
Age, years	70.0 (65.0–76.0)	67.5 (61.5–74.5)	71.0 (67.8–77.8)	0.033
Male, *n* (%)	50 (61.0)	28 (66.7)	22 (55.0)	0.392
BMI, kg/m^2^	23.8 ± 2.3	23.9 ± 2.3	23.6 ± 2.3	0.533
Stroke characteristics				
NIHSS baseline	12.0 (7.0–18.0)	8.0 (5.2–12.8)	15.5 (11.0–22.5)	<0.001
mTICI 1, *n* (%)	11 (13.4)	4 (9.5)	7 (17.5)	0.354
mTICI 2b, *n* (%)	43 (52.4)	21 (50.0)	22 (55.0)	—
mTICI 3, *n* (%)	28 (34.1)	17 (40.5)	11 (27.5)	—
Hematological parameters				
Neutrophil, ×10^9^/L	7.0 (5.9–8.5)	6.2 (5.6–8.0)	7.9 (6.6–9.3)	0.003
Lymphocyte, ×10^9^/L	2.5 (2.2–2.7)	2.6 (2.3–2.9)	2.3 (2.0–2.6)	0.003
Platelet, ×10^9^/L	241.0 (198.2–274.2)	238.5 (189.0–261.5)	242.5 (208.5–286.5)	0.268
Monocyte, ×10^9^/L	0.8 (0.7–1.0)	0.8 (0.7–0.9)	0.8 (0.7–1.0)	0.898
Inflammatory indices				
NLR	2.8 (2.2–3.9)	2.6 (2.0–3.3)	3.4 (2.5–4.6)	0.003
PLR	97.4 (75.9–118.9)	84.6 (72.9–105.0)	115.4 (83.5–122.3)	0.008
SII	673.4 (461.9–916.8)	552.0 (414.3–722.8)	782.9 (646.1–1,113.7)	0.001
SIRI	2.2 (1.8–3.5)	2.2 (1.7–3.1)	2.9 (1.9–4.7)	0.023

Values are median (IQR), mean ± SD, or *n* (%). *p* values from the Mann–Whitney *U* test (all continuous variables except BMI), Student’s *t*-test (BMI), or *χ*^2^/Fisher’s exact test (categorical). The following prognostic variables were not available in the dataset and could not be included: onset-to-reperfusion time, collateral status, infarct core volume, and hemorrhagic transformation.

### 3.4 mRS distribution by recanalization grade

**Figure 1:**
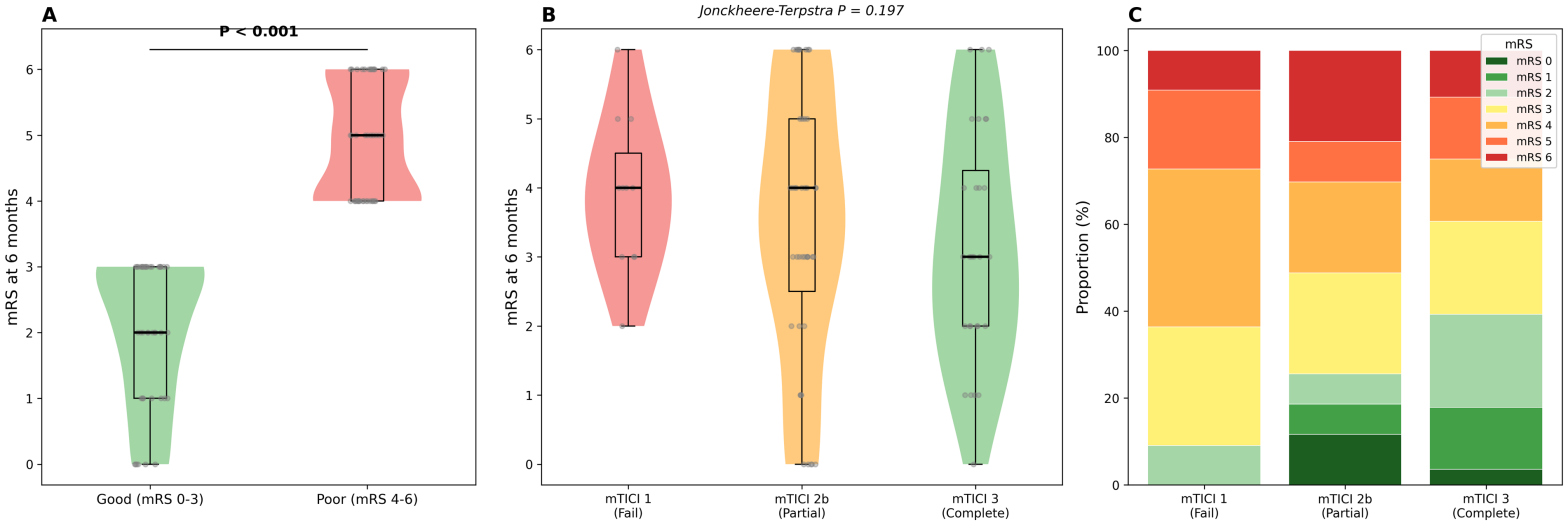
Distribution of 6-month mRS. **(A)** Violin plots of mRS within good (0–3) and poor (4–6) groups. **(B)** mRS distribution stratified by recanalization grade (mTICI 1, 2b, 3). **(C)** Stacked proportional bar chart of mRS across mTICI grades.

As shown in Figure 1, the proportion of poor outcomes decreased the proportion of poor outcomes decreased across recanalization grades (63.6% for mTICI 1, 51.2% for 2b, 39.3% for 3), although neither the Jonckheere–Terpstra (*p* = 0.197) nor the Cochran–Armitage (*p* = 0.149) trend test reached significance, likely owing to the small mTICI 1 subgroup (*n* = 11). This gradient is directionally consistent with the established prognostic importance of complete reperfusion after EVT (14). Notably, 39.3% of patients with complete recanalization still had poor outcomes, consistent with the concept of futile recanalization (15).

### 3.5 Logistic regression

**Figure 2:**
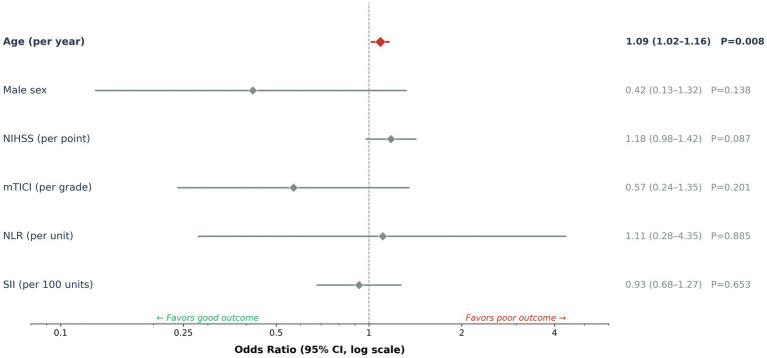
Forest plot of multivariable (Model 1) predictors of poor functional outcome. Only age was independently associated after adjustment.

As shown in Table 1 and Figure 2, in univariable analysis, age in univariable analysis, age (OR 1.07, 95% CI 1.01–1.13, *p* = 0.013), NIHSS (OR 1.14, 1.05–1.22, *p* < 0.001), NLR (OR 1.85, 1.21–2.83, *p* = 0.005), and SII (OR 1.20 per 100 units, 1.04–1.38, *p* = 0.010) were associated with poor outcome.

In Model 1 (age, sex, NIHSS, mTICI, NLR, SII), only age remained independently associated (OR 1.09, 95% CI 1.02–1.16, *p* = 0.008). NIHSS showed a non-significant trend (OR 1.18, 0.98–1.42, *p* = 0.087), and the inflammatory indices were clearly non-significant (NLR OR 1.11, *p* = 0.885; SII OR 0.93, *p* = 0.653). This pattern is the expected consequence of severe collinearity: NLR–NIHSS *r*_s_ = 0.88, with VIF = 8.7 for NLR and 5.6 for NIHSS in Model 1, inflating standard errors and rendering the individual NIHSS and inflammatory-index coefficients unreliable. The Hosmer–Lemeshow test indicated adequate calibration (*p* = 0.247).

We emphasize that a sensitivity model excluding NIHSS (Model 1 with NIHSS removed but all other covariates, including SII, retained), in which NLR reached nominal significance (OR 2.60, 95% CI 1.01–6.69, *p* = 0.048), cannot be interpreted as evidence of independent prediction. Removing NIHSS—the dominant known confounder and clinical reference standard—simply allows NLR to act as a noisy proxy for stroke severity. Indeed, the apparent effect of NLR collapses and reverses as soon as NIHSS is reintroduced (NLR OR 0.77, *p* = 0.564 in an NLR + NIHSS model), and the wide confidence interval (lower bound ≈ 1.0) reflects substantial imprecision. A Firth penalized regression (addressing potential small-sample bias and separation at the low EPV) yielded essentially unchanged coefficients: only age was robust, and NLR and SII remained non-significant, indicating that the null inflammatory-index estimates are not an artifact of small-sample bias or separation (though penalization does not, by itself, address statistical power). In a sensitivity analysis using the alternative mRS 0–2 vs. 3–6 dichotomy, the univariable associations of the inflammatory indices with poor outcome were unchanged in direction and remained significant (NLR OR 1.85, *p* = 0.023; SII OR 1.20 per 100 units, *p* = 0.034; SIRI OR 1.73, *p* = 0.028), confirming that the choice of outcome threshold did not drive the results.

### 3.6 Discrimination and incremental value

**Figure 3:**
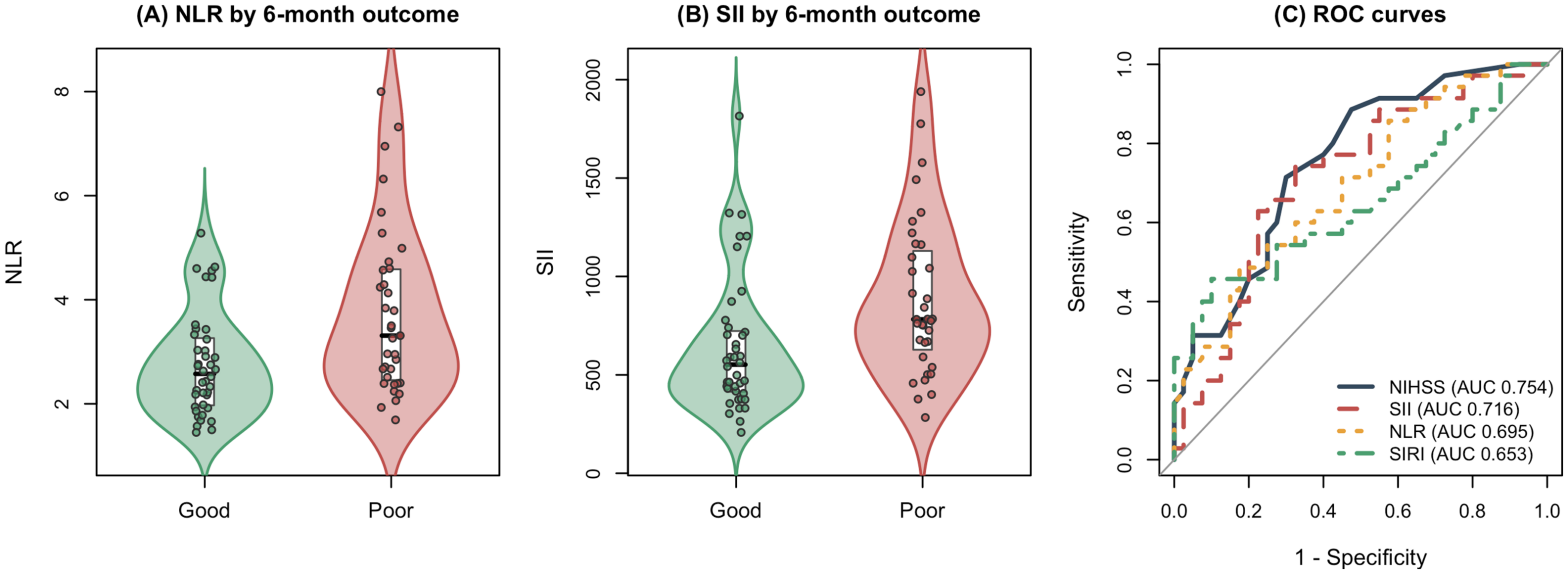
Inflammatory markers and discrimination for 6-month poor functional outcome (analysis cohort, *n* = 75). **(A,B)** Violin plots showing the distribution of NLR and SII in the good- and poor-outcome groups. **(C)** Receiver operating characteristic curves for NIHSS, NLR, SII, and SIRI; NIHSS showed the highest area under the curve (AUC 0.754). Panels are descriptive/discriminative and do not imply independent predictive value (see Table 3).

As shown in Table 3 and Figure 3, NIHSS had the highest discrimination for poor outcome (AUC 0.754, 95% CI 0.636–0.858), followed by SII (0.716, 0.592–0.836), NLR (0.695, 0.570–0.808), and SIRI (0.653, 0.514–0.781). In pairwise DeLong comparisons, NIHSS discriminated significantly better than SIRI (ΔAUC 0.101, *p* = 0.017), with a borderline advantage over NLR (ΔAUC 0.059, *p* = 0.069) and no significant difference from SII (ΔAUC 0.038, *p* = 0.41). The Youden-optimal cutoffs were NLR 3.5 (sensitivity 48.6%, specificity 82.5%) and SII 665 (sensitivity 74.3%, specificity 67.5%).

**Table 3:** Test for incremental value of inflammatory indices over NIHSS for poor functional outcome—no incremental value detected (*n* = 75).

Model	AUC (95% CI)	LRT vs. NIHSS, *p*	ΔAUC vs. NIHSS (DeLong *p*)	IDI (*p*)	Continuous NRI (*p*)
NIHSS (reference)	0.754 (0.636–0.858)	—	—	—	—
NIHSS + SII	0.754	0.702	−0.001 (0.91)	+0.001 (0.83)	−0.236 (0.33)
NIHSS + NLR	0.756	0.564	+0.002 (0.81)	+0.005 (0.49)	+0.100 (0.70)
NIHSS + SIRI	0.761	0.40	+0.006 (0.61)	+0.010 (0.34)	+0.200 (0.40)

Adding any inflammatory index to NIHSS produced no significant improvement in model fit (likelihood-ratio test), discrimination (ΔAUC), or reclassification (IDI, NRI), and increased the AIC in every case (ΔAIC +1.3 to +1.9). Decision-curve analysis (performed on the 76 patients with complete NLR and SII data) showed no meaningful net-benefit gain (mean net benefit over threshold probabilities 0.10–0.90: NIHSS 0.174, NIHSS + SII 0.177, NIHSS + NLR 0.173; Supplementary Figure S1). The NIHSS + SII model was adequately calibrated (Hosmer–Lemeshow *p* = 0.21) but showed no calibration improvement over NIHSS alone (*p* = 0.34). Confidence intervals around the incremental estimates were wide; these results indicate no detectable incremental value rather than proof of its absence. LRT, likelihood-ratio test; IDI, integrated discrimination improvement; NRI, net reclassification improvement.

Crucially, no inflammatory index showed detectable incremental value over NIHSS (Table 3). Adding SII, NLR, or SIRI to NIHSS did not significantly improve model fit (likelihood-ratio *p* = 0.70, 0.56, and 0.40, respectively) and increased the AIC in every case; the change in AUC was ≤ 0.006 and never significant (e.g., NIHSS vs. NIHSS + SII, ΔAUC −0.001, DeLong *p* = 0.91); the IDI was ≈0 (all *p* > 0.33) and the continuous NRI point estimates were small with confidence intervals spanning zero (negative for SII, −0.236). Decision-curve analysis showed no clinically meaningful net benefit from adding inflammatory indices across threshold probabilities of 0.10–0.90 (mean net benefit: NIHSS 0.174 vs. NIHSS + SII 0.177 vs. NIHSS + NLR 0.173; Supplementary Figure S1). These confidence intervals were wide (e.g., IDI 95% CI up to +0.03; continuous NRI spanning roughly −0.7 to +0.7), so these results are consistent with the null but, given the limited sample, do not establish the complete absence of a small incremental effect. The absence of detectable incremental value was also confirmed under the alternative mRS 0–2 vs. 3–6 endpoint (likelihood-ratio *p* = 0.70–0.86; AIC increased for every index; ΔAUC ≤ 0.006), indicating that this conclusion does not depend on the choice of outcome threshold.

### 3.7 Correlation between stroke severity and inflammatory indices

Baseline NIHSS correlated strongly with NLR (*r*_s_ = 0.88, *p* < 0.001), SIRI (*r*_s_ = 0.84), and SII (*r*_s_ = 0.78), and moderately with PLR (*r*_s_ = 0.48). Age was essentially uncorrelated with NIHSS (*r*_s_ = 0.08, *p* = 0.47) and with the inflammatory indices (|*r*_s_| < 0.16), confirming age as a prognostic dimension independent of stroke severity and inflammation.

### 3.8 Missing-data sensitivity analysis

Under multiple imputation (*m* = 25, all 82 patients with outcome data, Rubin’s rules; Supplementary Table S1), the conclusions of Model 1 were unchanged: age remained the only independent predictor (OR 1.08, 95% CI 1.02–1.15, *p* = 0.011), and the inflammatory indices remained non-significant (NLR OR 0.94, *p* = 0.93; SII OR 0.97, *p* = 0.86). If anything, NIHSS strengthened (OR 1.19, *p* = 0.055; OR 1.21, *p* = 0.044 in the R mice cross-check), reinforcing that stroke severity, not inflammation, is the operative predictor and indicating that complete-case exclusion did not bias our findings.

## 4 Discussion

### 4.1 Principal findings

In this exploratory single-center cohort, admission inflammatory indices (NLR, SII, SIRI, PLR) were associated with 6-month poor outcome after EVT at the univariable level, but this association did not survive adjustment for stroke severity: in multivariable analysis only age was independently associated with outcome, and formal testing showed no detectable incremental discriminative or clinical value beyond NIHSS. This pattern was consistent across likelihood-ratio, AUC, reclassification, decision-curve, penalized-regression, and multiple-imputation analyses, though the modest sample size means a small incremental effect cannot be formally excluded.

### 4.2 Comparison with prior literature

Our 48.8% rate of 6-month poor outcome and 15.9% mortality are concordant with the HERMES meta-analysis and major EVT trials (4, 16, 17). The univariable association of NLR with poor outcome aligns with meta-analytic data [Song et al. (18) pooled OR 2.49] and with EVT-specific reports (19). The relatively strong univariable performance of SII echoes studies linking SII to malignant edema and poor 3-month outcome after EVT (20, 21), and to symptomatic intracranial hemorrhage (22), while the weaker performance of SIRI—whose monocyte component may introduce additional variability (23)—mirrors reports in which SIRI lost significance after multivariable adjustment (24–26). Systemic inflammatory indices show broadly consistent prognostic signals across neurological injury more generally, including critical AIS and traumatic brain injury (27, 28), a pattern compatible with severity-coupled inflammation rather than an independent causal effect. Importantly, our contribution is not another positive association but a direct demonstration—within a single cohort and using formal incremental-value testing—that this association is largely a reflection of stroke severity. This nuance is frequently obscured when markers are reported in models that omit, or are dominated by, NIHSS.

### 4.3 Collinearity and the interpretation of “independence”

The strong NIHSS–NLR correlation (*r*_s_ = 0.88) reflects a fundamental biological coupling: severe ischemia drives a hypothalamic–pituitary–adrenal response causing neutrophil demargination and lymphocyte redistribution, so stroke severity predicts the magnitude of peripheral inflammation (29). Conversely, the resulting peripheral inflammation can itself aggravate ischemic injury through blood–brain barrier disruption, neutrophil extracellular trap formation promoting microvascular thrombosis, and complement-mediated neuronal injury (30–32), a bidirectional relationship that further entangles severity and inflammation. This coupling has a direct methodological consequence: when NIHSS and inflammatory indices are entered together, their coefficients become unstable and uninterpretable as independent effects (VIF 8.7 for NLR). It also explains why excluding NIHSS appears to “revive” NLR—the index merely substitutes for the omitted severity term. We therefore caution against the common practice of inferring independent prognostic value from severity-excluded models; the appropriate question is whether a marker adds value given NIHSS, which our incremental analyses answer in the negative. Notably, this conclusion rests on the incremental-value framework (likelihood-ratio, ΔAUC, NRI/IDI, decision curves)—which remains valid under collinearity—rather than on the non-significant individual coefficients of Model 1, whose interpretation collinearity precludes; the underlying structural fact (*r*_s_ = 0.88, VIF = 8.7) is itself independent of sample size.

### 4.4 Clinical implications

Our findings do not support the routine clinical use of inflammatory indices for prognostication after EVT, nor any change in management based on them. Because they add no detectable value beyond a NIHSS score that is already obtained at presentation, they cannot currently be recommended for risk stratification or treatment decisions. Their principal interest is mechanistic and hypothesis-generating: as low-cost, universally available markers, they may warrant prospective study of whether serial or dynamic inflammatory measures—rather than a single admission value—carry information not captured by baseline severity, and of whether anti-inflammatory adjuncts benefit high-inflammation subgroups within current management frameworks (33). Any role as a surrogate when NIHSS is unobtainable (e.g., intubated or aphasic patients, in whom standard stroke-scale assessment is unreliable) (34) remains speculative and entirely untested—our cohort did not include patients in whom NIHSS could not be assessed, so this possibility was not, and could not be, examined here.

### 4.5 Strengths and limitations

Strengths include the simultaneous head-to-head evaluation of four indices in a single EVT cohort, a 6-month outcome, blinded outcome assessment with documented reliability, and—most importantly—the explicit, formal testing of incremental value rather than reliance on association alone.

Limitations are substantial and constrain our conclusions to the exploratory. First, the single-center retrospective design and small sample (90 enrolled; 82 with follow-up; 75 with complete index data) limit generalizability and statistical power; the events-per-variable ratio (≈5.8) is below the conventional minimum, so multivariable coefficients should be regarded as imprecise (we mitigated, but cannot eliminate, this with penalized regression and multiple imputation). No *a priori* power calculation was performed. Importantly, the incremental-value analyses were correspondingly underpowered: the wide confidence intervals around the ΔAUC, IDI, and NRI estimates mean our negative findings should be interpreted as “no incremental value detected” rather than as proof that no small incremental effect exists. Second, inflammatory indices were measured at a single admission time point and cannot capture the dynamic inflammatory trajectory. Third, and critically, several established prognostic confounders were not available in our dataset and could not be adjusted for—including onset-to-reperfusion time, collateral status, infarct core volume, hemorrhagic transformation, and post-stroke complications—so residual confounding is likely and our estimates of association may be overstated. Fourth, the observational design precludes causal inference. Fifth, data-driven cutoffs were not externally validated. Sixth, the strong collinearity between NIHSS and the inflammatory indices fundamentally limits any assessment of their independent contribution. Multicenter prospective studies with serial biomarker sampling, comprehensive confounding adjustment, and external validation are required.

## 5 Conclusion

In patients undergoing EVT for anterior circulation AIS, admission systemic inflammatory indices (notably NLR and SII) are associated with 6-month poor functional outcome at the univariable level. However, this association is largely explained by stroke severity, and we found no detectable independent or incremental prognostic value beyond NIHSS by likelihood-ratio, AUC, reclassification, or decision-curve criteria, with findings unchanged under multiple imputation; the study was, however, underpowered to exclude a small incremental effect. These exploratory, hypothesis-generating results do not support clinical use of inflammatory indices for prognostication after EVT. Prospective, multicenter studies with serial measurements and comprehensive confounding adjustment are needed before any clinical role can be considered.

## Data Availability

The raw data supporting the conclusions of this article will be made available by the authors, without undue reservation.
